# ZnO/metal/ZnO (metal = Ag, Pt, Au) films for energy-saving in windows application

**DOI:** 10.1038/s41598-022-20043-8

**Published:** 2022-09-16

**Authors:** Mina Rabizadeh, Mohammad Hossein Ehsani, Mohammad Mahdi Shahidi

**Affiliations:** Faculty of Physics, Semnan University, Semnan, 35195-363 Iran

**Keywords:** Surfaces, interfaces and thin films, Applied physics

## Abstract

In this paper, the impact of different metals (Ag, Pt and Au) on the ZnO/metal/ZnO samples, being coated on a Glass substrate via RF/DC magnetron sputtering system, is investigated. The structural, optical, and thermal properties of the as-prepared samples were systematically studied for the purpose of storage and production of energy in industry. Our results show that these layers can be used as suitable coatings on building windows for energy storage applications. In the same experimental conditions, the case of Au as the intermediate layer has shown to have better optical and electrical conditions. Then the Pt layer also led to further improvement of the properties of the samples rather than those of the Ag. Moreover, the ZnO/Au/ZnO sample has shown the highest transmittance at the visible region (68.95%) and the highest FOM (5.1 × 10^–4^ Ω^−1^). Therefore, it can be considered as relatively the optimum sample in order for the building windows to save energy because of its low U-value (2.16 W/cm^2^ K) and low emissivity (0.45). Finally, by applying the equivalent voltage of 12 V at the ends of the sample, the surface temperature of sample has risen from 24 to 120 °C.

## Introduction

Low emission (low-E) transparent conducting oxides are integral to transparent conductive electrodes in the new generation of low-E optical-electrical devices, which are potential candidates for various applications such as flat panel displays, plasma screens, touch screens, organic light-emitting diodes, and solar cells. Nowadays, using such structures as energy-saving windows coating is prevalent.

Highly transparent low-E and heat reflective (TCOs) thin films have high transmission and reflection spectra in the visible and infrared ranges, respectively. These films can be used as coatings on building glass to save energy. Also, such samples are being applied as transparent conductive films in the industry, such as automotive glass, due to their remarkable low electrical resistance^1–3^. ITO has always been considered as a commonly used TCO in industry. Because of its fragility, toxicity, high cost, and the limited indium resources, researchers are looking for alternative materials^4,5^.

Due to the growing energy consumption worldwide, low-E materials are being extensively used. Glasses coated with low-E materials, for example, are applicable in buildings as windows or doors to decrease energy consumption. In summer, low-E films allow visible light to pass through and prevent IR waves from entering the building. In contrast, in winter, they avoid the infrared radiation emitted by heating devices in the building from passing to the outside. In other words, low-E films have a high transmittance in the visible region and high reflectance in the infrared region^6^.

Recent research has shown that three-layer conductive electrodes metal oxide/metal/metal oxide (O/M/O) have better electrical conductivity, optical resolution, and less emissivity than ITO films of the same thickness at room temperature. In those experiments, metal oxides such as ITO, ZnO, AZO, ZnS, WO_3_, MoO_3_, Nb_2_O_5_, and SnO_2_ as top and bottom layers, as well as metals including Ag, Cu, Ni, Al, Pt have been suggested to be applied as the intermediate layer^7–16^. Research has led to the improved electrical and optical properties in three-layer electrodes by changing the deposition conditions such as their temperature, pressure, bias voltage, etc. The choice of dielectric in the upper and lower layers and metal in the middle layer will be crucial in changing the optical and electrical properties. Moreover, ZnO has been widely applied in various industrial applications, including flat monitors, gas sensors, photosensors, and touchscreens^17^. Also, ZnO is highly regarded as an abundant, low-cost, non-toxic material that is stable against hydrogen plasma and high-temperature processes. For example, in 2012, Girtan et al. showed that ZnO/Ag/ZnO electrodes had better photovoltaic performance than ITO/Ag/ITO electrodes in solar cells^18^.

So far, the optical, electrical, and thermal properties of three metals of Au, Pt, and Ag in three-layer structures of ZnO/metal/ZnO, for use in energy storage coatings on building windows, have not been compared. In this study, we examined and compared the optical, electrical, and thermal properties of ZnO/metal/ZnO transparent conductive electrodes, considering the Ag, Au, and Pt metals in the middle layer, in the same deposition conditions for achieving the optimum condition. Also, these multilayer electrodes are compared with single ZnO electrodes. For this purpose, FESEM and RBS analyzes are performed to evaluate the thickness and concentration of the elements of the samples. Besides, by measuring the UV–Vis–NIR spectrum of each sample, the energy gap and their optical properties are examined; and finally, using the Window7.8 simulation software, the thermal properties of each sample are studied. To evaluate and compare the as-prepared samples for use in industry, we calculated three important parameters; emissivity, figure of merit, and U-value. When the glass absorbs heat or light energy, it is either transferred to the outside by air current or reflected by the surface of the glass. In a material, the capability of radiating energy is known as emissivity. Mostly, fenestration products emit or radiate heat (long-wave far-infrared energy), which is of significance in energy saving. Therefore, improving their insulating properties would be possible by decreasing their heat emission.

In this regard, the U-value has been suggested to be a good measure for the insulating quantity, which shows the rate of heat transfer in a fenestration product (W/m^2^ °C). This parameter includes conductive, convective, and radiative heat transfer of a system. As one can deduce, the smaller the U-value of a material, the lower the rate of heat flow would be. In addition, by calculating the total R-value, the thermal resistance of a sample can be represented, which is the reciprocal of the total U-value (R = 1/U)^19^.

## Experimental details

The ZnO sample was coated on a glass substrate by RF magnetron sputtering instrument (Nanostructured coatings co. DST3-T) deposition technique with the ZnO target (99.995% purity) with the RF power of 80 W. The substrate was situated at 75 mm distance from the target. ZnO deposition time in the top and bottom layers is 200 s with RF power. In the case of the middle layer, Au, Ag, and Pt metals with pure metal targets (99.995% purity) have been deposited at zero angles, and time deposition has been 40 s with DC power; the samples are nominated S_1_, S_2_, and S_3_, respectively.

Before each deposition, the chamber pressure reached 5 × 10^–3^ Torr, and metals and ZnO target were pre-sputtered for 5 min.

It is worth noting that the chamber has been flashed with Ar gas (99.995% purity) for three times to remove any contaminants. After preparing the samples, the four-point probe technique and Ultraviolet–Visible–Near IR (UV–Vis–NIR) spectrophotometer (JASCO V-670) were used to determine sheet resistance and optical properties of the samples, respectively.

To evaluate the elemental concentration and thickness of each layer, Rutherford backscattering spectrometry (RBS) analysis has been provided using a helium beam with 2 MeV energy. The experiment was performed on a 15° left lane line of Tehran Vandograph Laboratory in a conventional pixie chamber. A surface barrier detector at the angle of 165° was used to record the scattered particles. The reported data error related to fitting, cross-sectional data, beam energy, and incident load measurement is found to be 10%. The multilayer films’ total thickness was determined with field emission scanning electron microscope (FESEM) cross-section (Zeiss Sigma 300-HV).

## Results and discussion

### Rutherford backscattering analysis

**Figure 1:**
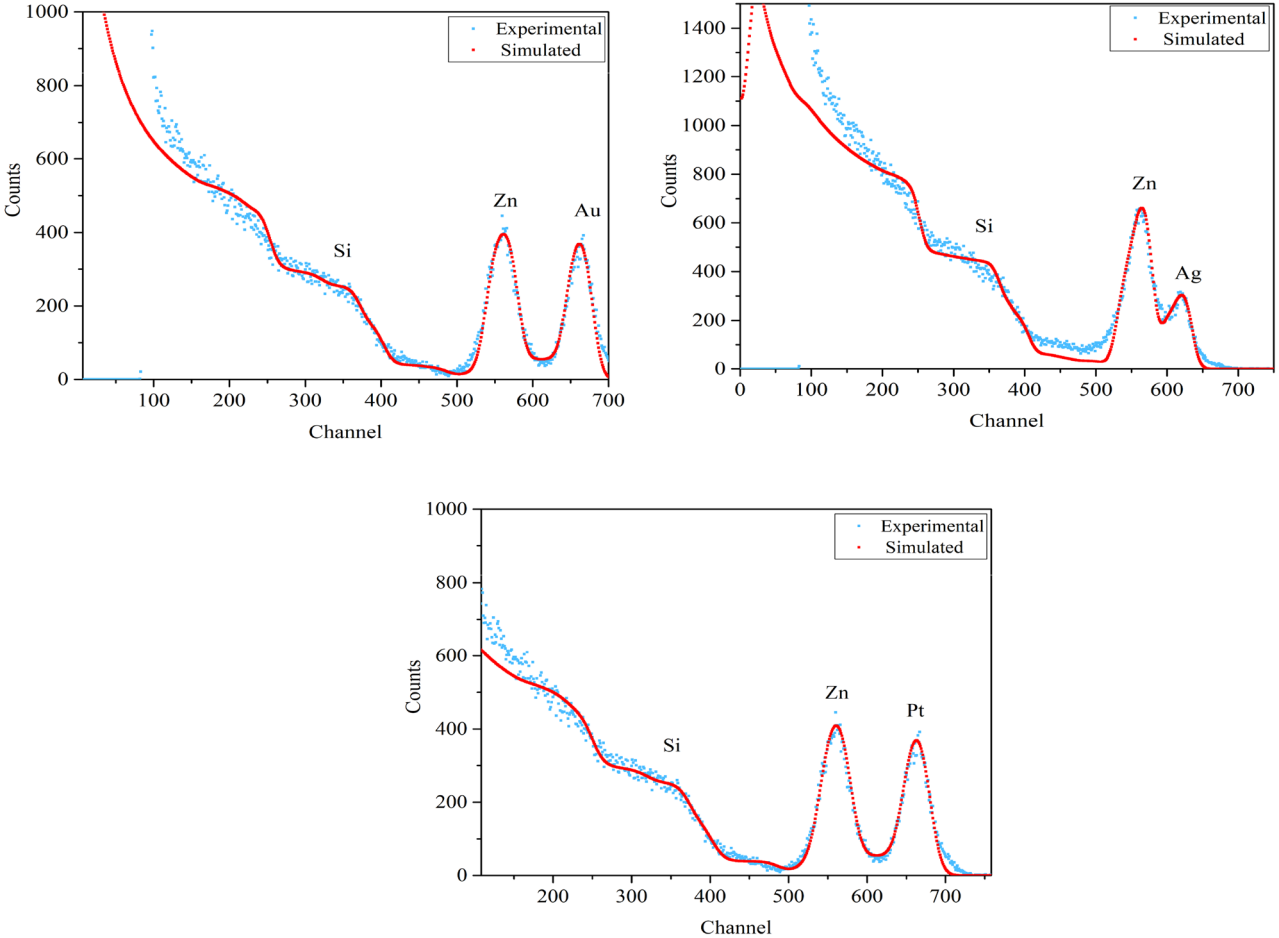
RBS spectra (experimental and simulation) of samples.

Figure 1 depicts 2 MeV RBS spectra of ZnO/metal/ZnO samples for different metals (Ag, Au, Pt). According to the shape of the diagrams simulated by SIMNRA software, they are well fitted with experimental data. As a result, the thickness and concentration of the material can be appropriately determined. These curves provide the backscattered energies of the incident particles for all samples. The channel region at 500–600 and 600–700 nm, respectively, contains a peak related to Zn, Au, Ag, and Pt metals. The valley’s depth between the Zn–Au and Zn–Pt signals is equal, and the valley between the Zn–Ag signals is reduced, while interlayer penetration is increased^20^. The thicknesses have been calculated in terms of monolayer (10^15^ atoms/cm^2^), corresponding to an areal atomic density and assuming uniform layer distribution. In the case that we consider the nominal stoichiometry of known atomic density (5.9 × 10^22^ atoms/cm^3^ for Au, 5.8 × 10^22^ atoms/cm^3^ for Ag, and 6.6 × 10^22^ atoms/cm^3^ for Pt), the thickness can be readily obtained in nm scale. The areal density of the Au, Ag, and Pt metals at the middle layer obtained from the RBS analysis are 25.5, 32.2 and 27.31 (× 10^15^ atoms/cm^2^), respectively. As a result, assuming the layer is uniform, the thickness of the Au, Ag, and Pt metal layers in the S_1_, S_2_ and S_3_ samples is are 4.08, 5.44 and 4.09 nm, respectively^21^.

### The FESEM cross-section

**Figure 2:**
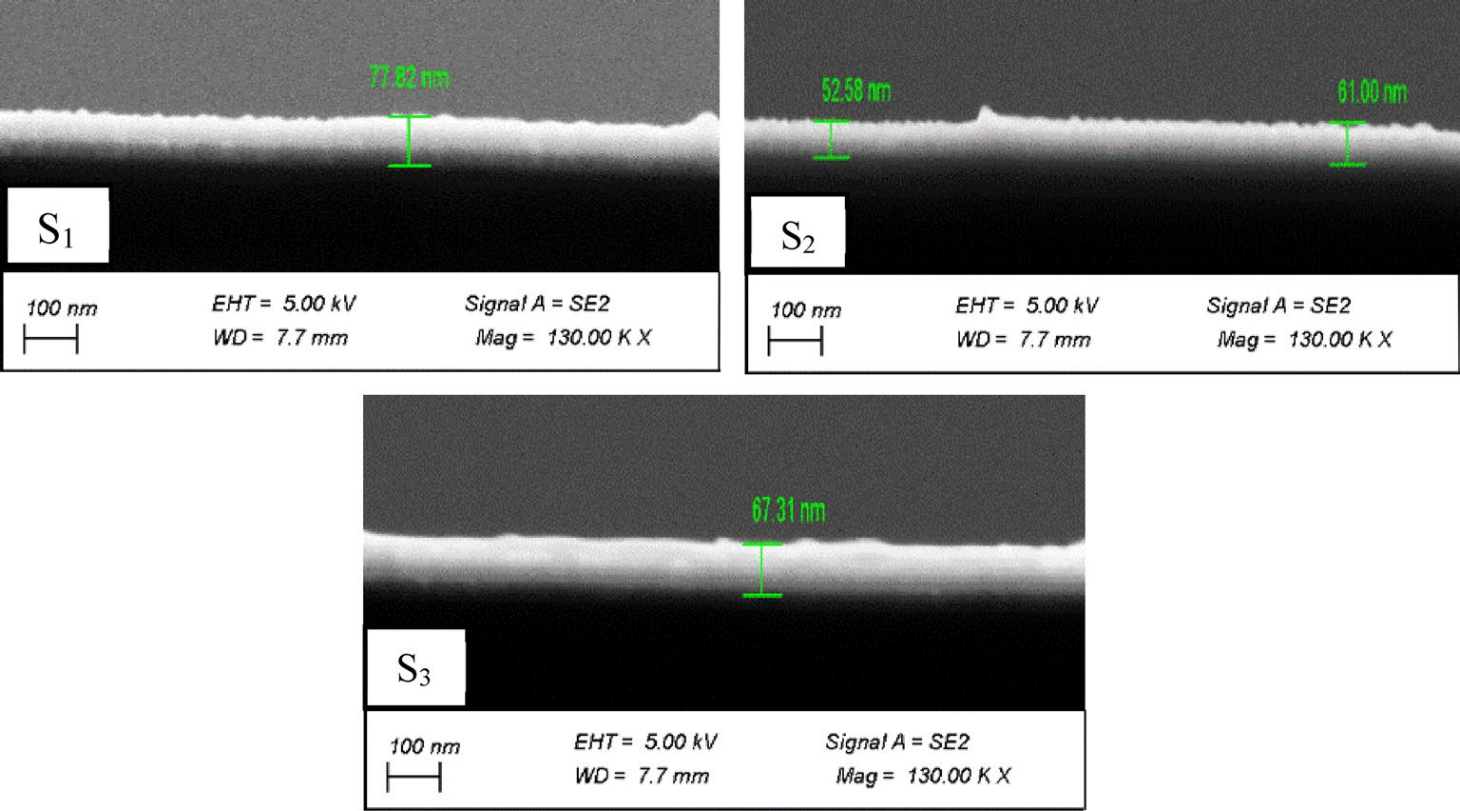
FESEM cross section diagram of samples.

Figure 2 shows the cross-section FESEM images of all samples. In the same experimental conditions, S_1_, S_2_, and S_3_ samples’ thicknesses are found to be 77, 61 and 63 nm, respectively.

### Calculation of sheet resistance

The sheet resistance of all the samples are measured using a four-point probe, which are shown in Table 1. The sheet resistance of the samples in the same conditions and duration of the coating indicates that the resistance of the ZnO single layer sample is very high. The presence of the middle layer of the metal reduces the electrical resistance. As shown in its strip structure, these structures can be considered connecting metal to a semiconductor. In the study of the structure of ZnO/metal, no barrier for transferring the electrons from the metal to the semiconductor is found after bonding; the electrons are readily transferred from the metal layer to the ZnO and conversely. In this case, the density of charge carriers is increased due to the injection of electrons from the metal into the semiconductor or conversely^20,22^. The sheet resistance of sample with Ag metal is higher than those of the samples coated with Au and Pt metals; while in most articles on three-layer structures, Ag metal was used in the middle layer, and low resistance is mentioned. Ag grows in Volmer–Weber (island) mode on oxide substrates. The high resistance in the sample with Ag metal is probably as a result of the presence of separate islands on the surface of Ag metal, and for the disappearance and cohesion of these islands, more Ag coating layer is required. According to the results obtained in our previous article^23^, at thicknesses below 10 nm, Ag is deposited as a discontinuous island. This indicates that having a three-layer structure with different metals requires more Ag than Au and Pt, which is more cost-effective.

**Table 1:** Sheet resistance of samples.

Samples	Sheet resistance (Ω/sq)
ZnO	4.43 × 10^6^
S_1_	47.03
S_2_	358.59
S_3_	55.74

### Calculation of transmittance, reflectance, and average transmittance

**Figure 3:**
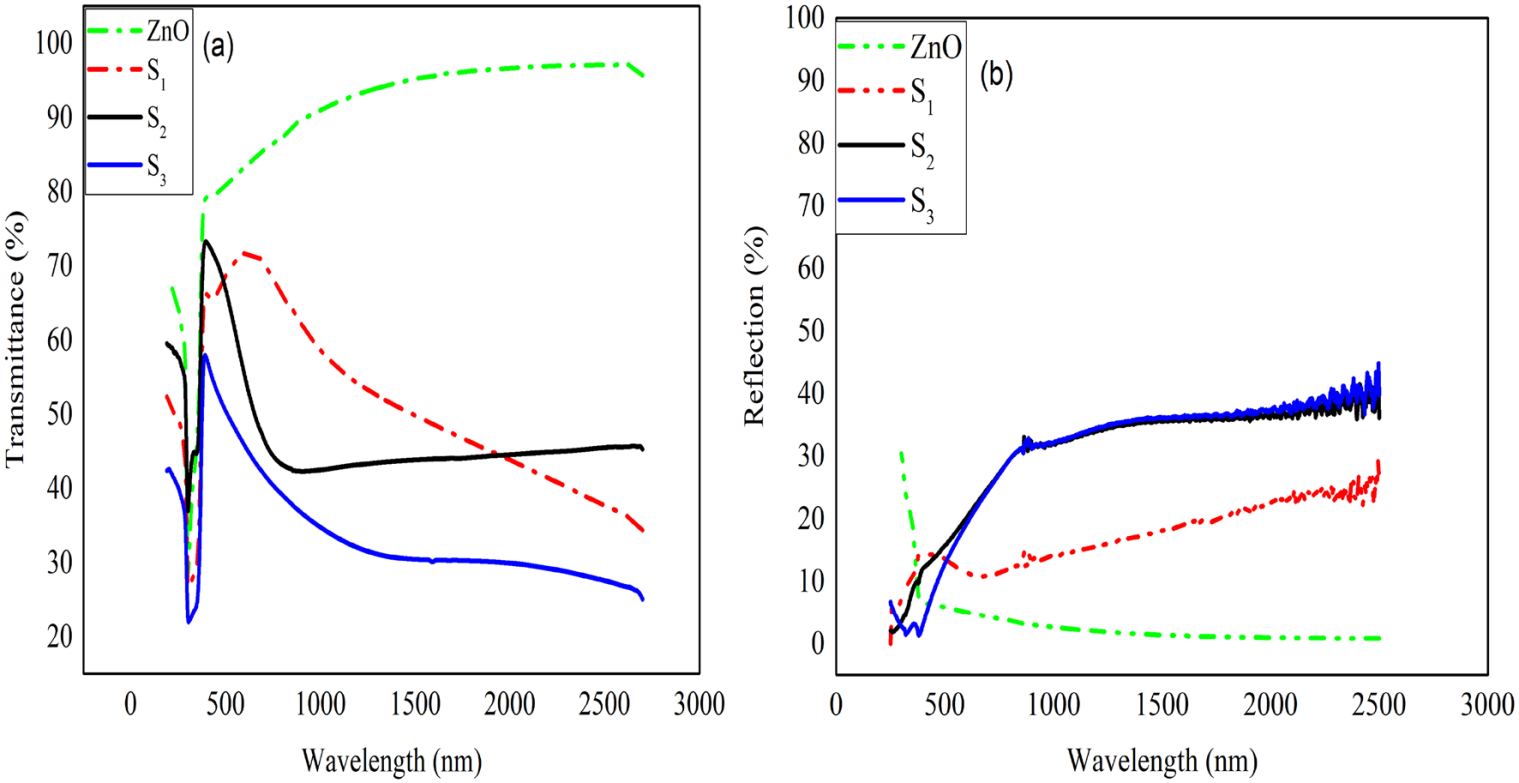
The transmittance and reflection spectra.

The transmission and reflection spectra of samples have been provided in the wavelengths of 190–2700 nm, shown in Fig. 3. In Fig. 3a, which is related to the transmittance diagram of the samples, the peaks of the sample with different metals are located at different wavelengths; so that the peaks of S_1_, S_2_, and S_3_ are at 626, 400 and 380 nm and have a transmittance of 71, 72 and 57%, respectively.

Due to the importance of the transmission in the range of 400–800 nm in industry and its better comparison for samples with different metals, the transmission coefficient (T_av_) for each sample is calculated for visible (T_vis_), solar (T_solar_), and NIR (T_NIR_) region, shown in Table 2.

**Table 2:** Average transmittance and reflectance of samples in visible, solar, and NIR regions.

Samples	T_solar_ (%)	R_solar_ (%)	T_NIR_ (%)	R_NIR_ (%)	T_vis_ (%)
S_1_	53.57	14.60	50.64	13.55	68.95
S_2_	50.78	24.09	43.76	22.53	58.76
S_3_	37.28	23.14	31.77	21.34	47.54

The transmission coefficient is obtained in the following:

1 $$ T_{av} = \frac{{\int {T(\lambda )V(\lambda )d\lambda } }}{{\int {V(\lambda )d\lambda } }}, $$

where V(λ) and T(λ) are, respectively, the luminous spectral efficiency and the transmission of the samples^19,23^.

The T_vis_ values of the S_1_, S_2_, and S_3_ samples are 68.95, 58.76 and 47.54%, respectively; the highest value belongs to the S_1_ sample.

The comparison of the reflection in the IR region for three-layer ZnO/metal/ZnO electrodes considering different metals in the same fabrication process has not been investigated. Infrared reflectance is one of the most crucial parameters of electrodes for use in industry. In this study, the comparison of the reflectance of these electrodes in the IR region was investigated for the first time.

According to Fig. 3b, the reflection of the S_1_, S_2_, and S_3_ samples in the near-infrared region is equal to 19, 35 and 36% at a wavelength of 1700 nm, respectively. ZnO single layer has the lowest reflectance in this range. Table 2 shows the average reflection in the solar and near-infrared regions, which is calculated using Eq. (1), with the difference that the amount of reflection in the solar and infrared regions has been replaced by the amount of transmission.

### Calculation of FOM parameter

Both conductivity and transparency parameters are very critical in the industry. For a better comparison of the properties of metal-oxide/metal/metal-oxide three-layer electrodes, the figure of merit parameter is used, which can be calculated from the following equation^24^.

2 $$ FOM = \frac{{T_{av}^{10} }}{{R_{sh} }}. $$

The larger the amount of FOM, the more transparent the conductive electrode will be. The FOM values of the samples are shown in Table 3. According to the mentioned values, the maximum amount of FOM is related to the S_1_ sample with the value of 5.1 × 10^–4^ Ω^−1^.

**Table 3:** Emissivity, FOM, and U-value of all samples.

Samples	ε_1_	ε_2_	Figure of merit × 10^–4^ (Ω^−1^)	U-value (W/m^2^ K)
S_1_	0.45	0.49	5.1	2.161
S_2_	3.98	3.80	0.136	2.567
S_3_	0.51	0.59	0.105	2.229

### Calculation of emissivity

Transparent conductive electrodes with low emissivity characteristics are a razor-thin, colorless, non-toxic coating that are used in window glass to enhance energy efficiency. Such windows are remarkably safe and are becoming standardized from the energy efficiency point of view in the modern home. Low-E windows prevent infrared light from penetrating into the glass from the outside. Moreover, these windows keep heating/cooling energy. The emissivity is dependent of the R_sh_ and is obtainable in the wavelength range of 780–2700 nm using the following equation^6,19,20^

3 $$ \varepsilon_{1} = 0.0129R_{sh} - 6.7 \times 10^{ - 5} R_{sh}^{2} . $$

Besides that, for samples having the equivalent wavelengths of λ > 3 µm and R_sh_ ˂˂ Z_0_ are also obtainable as follow:

4 $$ \varepsilon_{2} = \frac{{4R_{sh} }}{{Z_{0} }}, $$

where Z_0_ is the impedance of the vacuum (377 Ω). The acquired data of emissivity for all the samples are shown in Table 3. The minimum amount of emissivity is related to the S_1_ sample with the value of 0.45.

### Calculation of bandgap energy (E_g_)

**Figure 4:**
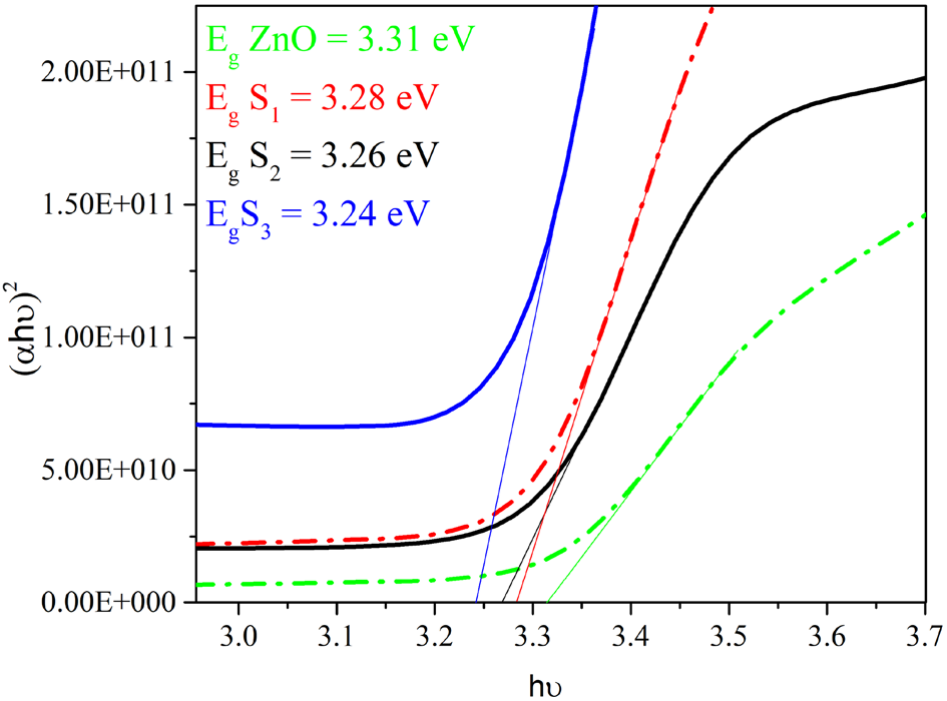
Optical bandgap energy of all samples.

The absorption coefficient (α) has been obtained for the direct transition using the following equation^25^:

5 $$ (\alpha h\nu )^{2} = A(hv - E_{g} ), $$

where h and A represent the incident radiation energy and a constant, respectively. The direct E_g_ is acquired by extrapolating the linear parts of the plots to zero absorption (αhυ = 0). The E_g_ changes for the ZnO/metal/ZnO multilayer system is provided in Fig. 4. According to the results obtained from the calculation of the bandgap energy, the energy gap of the ZnO sample is equal to 3.31 eV, which decreases for the ZnO/M/ZnO (M = Au, Ag, and Pt) thin-film samples. These bandgap changes in three-layer structures with different metals are consistent with the transmission changes in the visible region, so that the transmittance of ZnO single layer was maximum, and it can be seen that the band gap for this sample is also maximum. Also, the transmittance of the S_1_ sample is higher than that of the S_2_ sample, and the bandgap of the S_3_ sample is minimal^26^.

### Heating results

**Figure 5:**
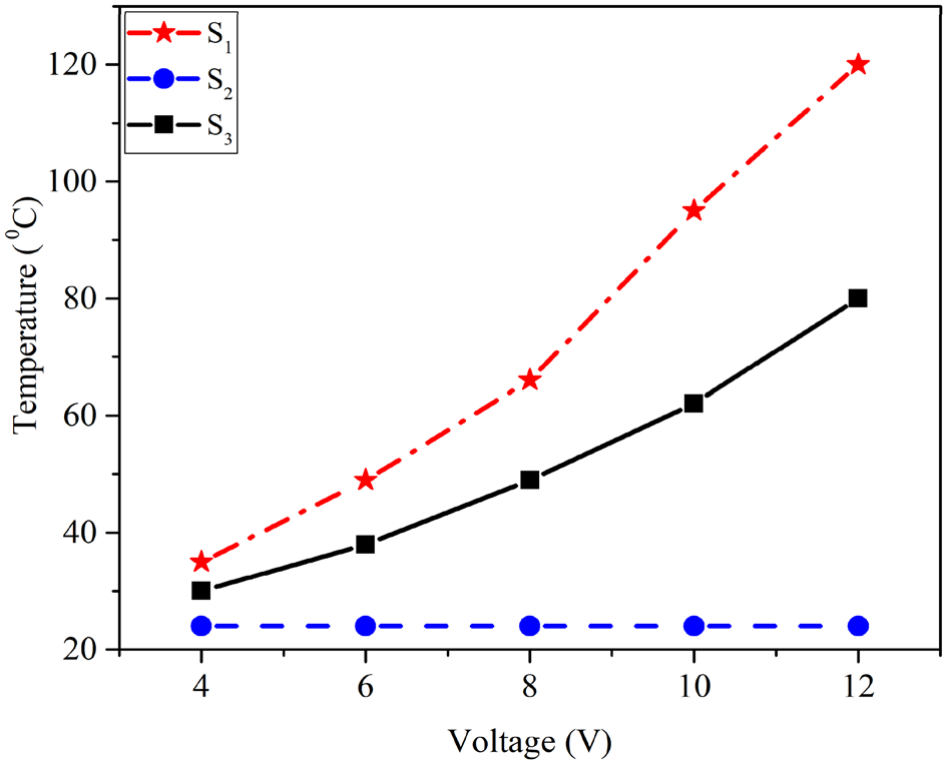
Temperature of the ZnO/metal/ZnO multilayer-based thin film heater depending on input DC voltage.

For studying the heating impacts of samples, their electro-thermal behavior is examined and shown in Fig. 5. For this purpose, silver contacts have been coated on both sides of the samples by an electron beam evaporator. Then, by applying a certain voltage for 300 s, the maximum temperature of produced heat between the contacts was measured by a thermal camera. According to Fig. 5, the temperature of the S_1_ sample, with a voltage change from 4 to 12 V, has a sharp increase in temperature from 35 to 120 °C, while this temperature increment is found to be less for the S_3_ sample (from 30 to 80 °C), and the S_2_ sample did not show any temperature change with increasing voltage.

Here, the heat loss as a result of the conduction and radiation from the back can be neglected since the glass substrate is not a good thermal conductor. Therefore, the prominent path of heat loss, which is air convection, is obtainable according to the following formula^27^:

6 $$ \begin{gathered} \Delta Q_{g} = P\Delta t = \frac{{V^{2} }}{R}\Delta t = Q_{conv} = h_{conv} A_{conv} (t_{s} - t_{i} ) \hfill \\ t_{s} = \frac{{V^{2} \Delta t}}{{Rh_{conv} A_{conv} }} + t_{i} . \hfill \\ \end{gathered} $$

Q_g_ is heat generated at power P for a time duration of Δt, h_conv_ is the convective heat transfer coefficient, A_conv_ is the surface area, and t_s_ and t_i_ are the saturation and initial temperatures, respectively. As one can infer, with the voltage increment and the resistance decrement, the saturation temperature increases. The S_1_ sample has less sheet resistance and more h_conv_ than the S_3_ sample, and for this reason, it can be seen in Fig. 5 that the amount of heat production in the S_1_ sample at a specific voltage is significantly increased compared to that of the S_3_ sample. The temperature of the S_2_ sample, probably due to its high resistance, did not decrease below 4 to 12 V.

### Thermal performance

To estimate the amount of heat passing through the material, the T_vis_, R_vis_, T_solar_, R_solar_, T_NIR_, R_NIR_ and emissivity values of the samples must be calculated^28^. The T_vis_ and R_vis_ are the rate of transmission and reflection in the area of 400 < λ < 800 nm, T_solar_, R_solar_ are the rate of transmittance and reflection in the area of 250 < λ < 2500 nm, and T_NIR_, R_NIR_ are the rate of transmittance and reflection in the area of 780 < λ < 2500 nm; all of which are shown in Table 2. In this study, to calculate the U-value of each sample, using Window7.8 software, we simulated a double-glazed system comprised of two layers of glass with a thickness of 4 mm and a gap layer containing argon gas. The U-values of all samples are listed in Table 3. In the absence of the ZnO/metal/ZnO coating on double-glazed windows, the U-value is 2.730 W/m^2^ K. However, after the deposition process of the transparent conductive electrode coating, the U-value decreased significantly. The S_1_ (ZnO/Au/ZnO) sample has the lowest, and the S_2_ (ZnO/Ag/ZnO) sample has the highest U-value due to its high sheet resistance and high emissivity.

## Conclusion

In this work, the structural, optical, electrical, and thermal properties of ZnO/metal/ZnO three-layer films are studied, in which Au, Ag, and Pt metals are deposited in the middle layer on glass substrate using the magnetron sputtering technique. The structural properties of the samples have been studied using RBS and FESEM analyzes. In addition, the optical properties of the material were investigated by measuring their transmission and reflection spectrum in the range of 190–2700 nm. The lowest amount of resistance before and after annealing is related to the ZnO/Au/ZnO sample, followed by the highest amount of heat production by applying voltage. The transmittance and reflectance spectra of the samples show that the highest values of the average transmittance in the visible region and the reflectance at the wavelength of 1700 nm are related to the ZnO/Au/ZnO and ZnO/Pt/ZnO samples, respectively. Also, the FOM values in the ZnO/Au/ZnO sample has a maximum value of 5.1 × 10^–4^ Ω^−1^. The FOM values of ZnO/Ag/ZnO and ZnO/Pt/ZnO samples are 0.136 × 10^–4^ and 0.105 × 10^–4^ (Ω^−1^), respectively. The amounts of emissivity, T_av_, and R_1700_ are very effective in heat transfer rate through the material (U-value). For example, the ZnO/Au/ZnO sample has the lowest U-value of 2.16 W/m^2^ K. According to the provided analyzes and calculations, ZnO/Au/ZnO and ZnO/Pt/ZnO samples have a better performance in heat production by applying voltage and reducing the transfer of heat through matter and emissivity than ZnO/Ag/ZnO.

## Data Availability

The datasets used and analyzed during the current study available from the corresponding author on reasonable request.
